# Evaluation of Prediction Models for Type 2 Diabetes Relapse After Post-bariatric Surgery Remission: a Post hoc Analysis of 15-Year Follow-up Data from the Swedish Obese Subjects (SOS) Study

**DOI:** 10.1007/s11695-020-04763-2

**Published:** 2020-06-13

**Authors:** Kajsa Sjöholm, Per-Arne Svensson, Magdalena Taube, Peter Jacobson, Johanna C. Andersson-Assarsson, Lena M. S. Carlsson, Markku Peltonen

**Affiliations:** 1 Institute of Medicine, Sahlgrenska Academy, University of Gothenburg, Gothenburg, Sweden; 2 Institute of Health and Care Science, Sahlgrenska Academy, University of Gothenburg, Gothenburg, Sweden; 3 Department of Neurobiology, Care Sciences and Society, Karolinska Institutet (Solna), Stockholm, Sweden; 4 Public Health Promotion Unit, National Institute for Health and Welfare, Helsinki, Finland

**Keywords:** Bariatric surgery, Type 2 diabetes relapse, Prediction model, Type 2 diabetes mellitus, Weight reduction

## Abstract

**Purpose:**

Many patients achieve type 2 diabetes (T2D) remission after bariatric surgery, but relapse after post-surgery remission is common. Scoring models accurately predict remission up to 5 years after surgery but have not been tested for prediction of long-term T2D relapse. The aim of this work was to test the ability of prediction models and single predictors to identify patients at risk of long-term relapse (10–15 years) after post-surgery T2D remission.

**Methods:**

We identified 222 individuals with T2D from the surgically treated group in the prospective Swedish Obese Subjects study, who were in remission at the 2-year follow-up and had data available for prediction of long-term T2D relapse. T2D remission/relapse was assessed after 10 and 15 years. Model performance (discrimination) was evaluated by the area under the receiver operating characteristic (AUROC) curves.

**Results:**

Preoperative prediction of relapse using scores DiaRem, Ad-DiaRem, and DiaBetter and T2D duration alone was poor, as indicated by AUROC curves between 0.61–0.64 at 10 years and 0.62–0.66 at 15 years. Likewise, the 5y-Ad-DiaRem score, which includes early postoperative measures, resulted in AUROC curves of 0.65 and 0.70 for relapse at 10 and 15 years, respectively. Two-year weight change alone had higher discriminatory capacity than the 5y-Ad-DiaRem model at 10 years (AUROC = 0.70; *p* = 0.036) and similar capacity at 15 years (AUROC = 0.78; *p* = 0.188).

**Conclusions:**

Predictive performance of all tested models is low for T2D relapse. By contrast, a single measure of 2-year weight change after surgery was associated with relapse, supporting a key role for initial weight reduction in long-term T2D control.

**Electronic supplementary material:**

The online version of this article (10.1007/s11695-020-04763-2) contains supplementary material, which is available to authorized users.

## Introduction

Type 2 diabetes (T2D) is a chronic disease, associated with severe macrovascular and microvascular complications [1]. Bariatric surgery is the most effective treatment for achieving sustained weight loss, and T2D remission after surgery is common but not always persistent long term [2, 3]. A reliable risk prediction model for T2D relapse after initial remission could be used to identify patients who should be targeted for post-surgery intervention and medical management to reduce the risk of relapse and prevent T2D complications.

Using retrospective data, preoperative scoring models (DiaRem, Ad-DiaRem, DiaBetter) have been developed and validated to accurately predict T2D remission up to 5 years after gastric bypass surgery [4–10]. However, preoperative prediction of T2D relapse after initial remission seems more challenging and it has been suggested that in addition to preoperative measures including T2D duration and glycemic control, postoperative factors, such as weight trajectories, also need to be considered when optimized prediction models are developed [11, 12]. Indeed, the 5y-Ad-DiaRem scoring model includes several postoperative measures including weight reduction and T2D status 1 year after surgery, and is suggested to better identify patients at risk of T2D relapse over 5 years [12]. Other studies have also identified early weight change as a relapse predictor [11–13] and in line with these reports, we previously showed that patients with a large 2-year weight loss were less likely to relapse between 2 and 10 years of follow-up in the prospective Swedish Obese Subjects (SOS) study [2]. However, whether preoperative or postoperative scoring models are useful tools for prediction of T2D relapse over the long term has not been assessed nor compared with single predictors such as baseline T2D duration or short-term weight change.

Thus, the objective of this report was to evaluate the predictive capacities of existing scoring models and previously identified independent predictors for T2D relapse over 10 and 15 years of follow-up in patients with T2D remission after bariatric surgery.

## Methods

### Study Design

The SOS study has previously been described [2, 3, 14]. The study was approved by the relevant ethical review boards and informed consent was obtained from all participants. In brief, 2010 individuals chose to undergo surgery, and a matched control group with obesity consisting of 2037 participants was created using 18 variables. Three patients never underwent surgery and the per-protocol surgery group thus consists of 2007 participants. The inclusion and exclusion criteria were identical for the two study groups, and all participants were eligible for surgery (Supplementary Table 1). The inclusion criteria were aged 37 to 60 years and BMI of 34 kg/m^2^ or more for men and 38 kg/m^2^ or more for women before or at the matching examination. The exclusion criteria were established to exclude patients with unacceptable surgical risks. The type of surgery was determined by surgeons at the participating surgical departments. Patients were recruited between September 1, 1987, and January 31, 2001, and followed with physical examinations and questionnaires at baseline and after 0.5, 1, 2, 3, 4, 6, 8, 10, and 15 years.

Blood samples were taken after an overnight fast at baseline and after 2, 10, and 15 years. HbA1c and C-peptide levels were analyzed at the St. Vincent’s Healthcare Group, Dublin, Ireland, accredited by the Irish National Accreditation Board (registration number: 192MT in compliance with ISO/IEC 15189:2012). All other measurements were analyzed at the Central Laboratory, Sahlgrenska University Hospital, Gothenburg, Sweden, accredited according to ISO=International Organization for Standardization 15189:2007 standards. From 1987 through 2009, fasting glucose concentrations were measured in venous whole blood. After 2009, venous fasting plasma glucose was measured, and the concentrations were converted to those for blood glucose [15]. The SOS study was started before repeated measurements were routinely used for the diagnosis of T2D [16], and single determinations of fasting glucose or HbA1C were therefore used. Type 1 diabetes and latent autoimmune diabetes of adults in patients with diabetes onset before age 35 years were ruled out by excluding patients positive for glutamate decarboxylase antibodies or islet cell antibodies or with C-peptide values lower than 1.11 ng/mL at baseline. Two patients in the surgery group were excluded [14]. Self-reported medication and T2D duration was obtained from SOS questionnaires. T2D was a prespecified secondary end point.

### Prediction Models

The preoperative DiaRem, Ad-DiaRem, and DiaBetter scores and the postoperative 5y-Ad-DiaRem score were calculated essentially as previously described (Supplementary Table 2). Preoperative scores include various combinations of parameters such as baseline T2D duration, number of antidiabetic treatments, and HbA1C levels [4, 6, 7]. The 5y-Ad-DiaRem score combines preoperative data with postoperative 1-year data on blood glucose levels, number of antidiabetic treatments, remission status, and percent weight loss [12]. In the SOS study, T2D status was not determined at the 1-year follow-up, and therefore 2-year data were used for calculation of the 5y-Ad-DiaRem. Predictive capacity of composite models was compared with T2D duration and 1- and 2-year weight change as single predictors.

### Type 2 Diabetes–Related Definitions

Baseline T2D and 10- or 15-year relapse were defined as HbA1c ≥ 48 mmol/mol (6.5%) or blood glucose of ≥ 6.1 mmol/L (plasma glucose ≥ 7 mmol/L), or T2D medication use (insulin, oral antidiabetic drugs, or both). Remission of T2D at the 2-year follow-up was defined as HbA1c < 48 mmol/mol or blood glucose concentration < 6.1 mmol/L and no T2D medication.

### Statistical Analyses

Mean values, standard deviations, and percentages were used for describing study population characteristics. Differences between groups were tested with a *t* test for continuous and Fisher’s exact test for dichotomous variables. Prediction performance in terms of discrimination was evaluated by calculating the area under the receiver operating characteristic (AUROC) curves. Data were analyzed per-protocol; i.e., patients who underwent surgery to restore normal anatomy were treated as censored observations on the corresponding date. A two-sided *p* value < 0.05 was considered statistically significant. All statistical analyses were performed using Stata (version 15.1).

## Results

### Baseline Characteristics and Follow-up Relapse Rates

The current analyses include 222 patients from the SOS surgery group who were in remission at the 2-year follow-up and had available preoperative and 2-year postoperative data. Of these, *n* = 132 underwent vertical banded gastroplasty, *n* = 45 underwent nonadjustable or adjustable banding, and *n* = 45 underwent gastric bypass surgery.

Preoperative and postoperative characteristics of study participants stratified by non-relapse and relapse, 10 and 15 years after bariatric surgery, are presented in Table 1. The overall relapse rates were 54% (95% CI 46–62%) at 10 years and 61% (95% CI 52–70%) at 15 years. At baseline, patients who relapsed between 2 and 10 years or between 2 and 15 years had lower BMI, higher preoperative scores, and higher levels of blood glucose. The baseline T2D duration was 0.4 and 0.3 years, respectively, in non-relapse and 1.1 and 0.9 years, respectively, in relapse groups at 10 and 15 years.

**Table 1:** Preoperative and postoperative characteristics of SOS study patients who were in remission after 2 years: data shown for full cohort, and stratified by type 2 diabetes status after 10 and 15 years of follow-up

	All (*N* = 222)^†^	10-year status (*N* = 169)			15-year status (*N* = 111)		
		Non-relapse	Relapse	*p* value	Non-relapse	Relapse	*p* value
Preoperative (baseline)							
*N*	222	78	91		43	68	
Women	138 (62.2%)	53 (67.9)	48 (52.7)	0.059	28 (65.1)	32 (47.1)	0.079
Age (years)	48.6 (5.9)	48.2 (6.2)	49.3 (5.4)	0.215	48.3 (6.5)	48.8 (5.2)	0.685
Body mass index (kg/m^2^)	42.8 (4.9)	43.8 (5.7)	41.9 (4.2)	0.016	44.7 (6.3)	42.4 (4.9)	0.034
HbA1c (mmol/mol)	57.4 (15.2)	54.7 (14.9)	58.8 (15.1)	0.076	52.2 (13.5)	58.7 (14.2)	0.017
HbA1c (%)	7.4 (1.4)	7.2 (1.4)	7.5 (1.4)	0.076	6.9 (1.2)	7.5 (1.3)	0.017
Blood glucose (mmol/L)	7.5 (2.4)	7.2 (1.9)	7.9 (2.6)	0.039	6.9 (1.9)	7.6 (2.4)	0.068
Insulin (mU/L)	27.8 (13.2)	29.9 (13.6)	26.7 (11.8)	0.113	31.8 (17.1)	27.9 (13.0)	0.167
C-peptide (ng/mL)	4.8 (1.4)	4.8 (1.3)	4.7 (1.2)	0.622	5.1 (1.5)	4.6 (1.2)	0.058
T2D duration at baseline (years)	0.9 (1.5)	0.4 (1.1)	1.1 (1.6)	0.002	0.3 (0.9)	0.9 (1.5)	0.018
Screen-detected T2D at baseline	149 (67.1)	65 (83.3)	51 (56.0)	< 0.001	37 (86.0)	42 (61.8)	0.009
Smoke daily	51 (23.0)	19 (24.4)	20 (22.0)	0.719	11 (25.6)	18 (26.5)	1.000
DiaRem score	4.8 (3.0)	4.1 (2.6)	5.3 (3.0)	0.007	3.7 (2.5)	5.3 (3.1)	0.005
Ad-DiaRem	5.2 (2.8)	4.6 (2.7)	5.7 (2.7)	0.010	4.4 (2.7)	5.7 (2.9)	0.017
DiaBetter	1.9 (1.8)	1.4 (1.7)	2.1 (1.7)	0.007	1.3 (1.4)	2.1 (1.8)	0.015
Postoperative (2 years)							
Body mass index (kg/m^2^)	32.2 (4.7)	30.9 (4.8)	33.6 (3.9)	< 0.001	30.5 (5.5)	33.4 (4.0)	0.001
HbA1c (mmol/mol)	39.0 (4.1)	37.6 (4.0)	40.1 (4.0)	< 0.001	36.7 (4.3)	40.0 (4.0)	< 0.001
HbA1c (%)	5.7 (0.4)	5.6 (0.4)	5.8 (0.4)	< 0.001	5.5 (0.4)	5.8 (0.4)	< 0.001
Blood glucose (mmol/L)	4.4 (0.6)	4.1 (0.6)	4.5 (0.6)	< 0.001	4.0 (0.5)	4.5 (0.6)	< 0.001
Insulin (mU/L)	11.2 (6.5)	10.0 (7.7)	12.9 (5.8)	0.005	10.0 (8.6)	12.4 (6.3)	0.082
Smoke daily	57 (26.3)	25 (32.1)	19 (21.3)	0.159	11 (25.6)	21 (31.3)	0.667
5y-Ad-DiaRem score	2.8 (2.1)	2.2 (2.1)	3.2 (1.9)	0.002	1.8 (1.8)	3.1 (2.0)	0.001
Relative weight change (%)	− 24.4 (10.3)	− 29.1 (10.4)	− 19.8 (7.6)	< 0.001	− 31.1 (11.2)	− 20.9 (8.4)	< 0.001

Missing values at baseline: blood glucose, *n* = 1; C-peptide, *n* = 2; smoking status, *n* = 5. Data are mean (SD) or *N* (%). *T2D*, type 2 diabetes

At the 2-year follow-up, patients who later relapsed displayed higher BMI, higher levels of HbA1c and blood glucose, and higher 5y-Ad-DiaRem scores. Furthermore, patients who later relapsed had on average lost less weight at the 2-year examination compared with patients who were in remission at 10 and 15 years. Patients in non-relapse groups had similar baseline age and levels of insulin compared with relapse groups.

### Preoperative Prediction of Type 2 Diabetes Relapse

**Fig. 1:**
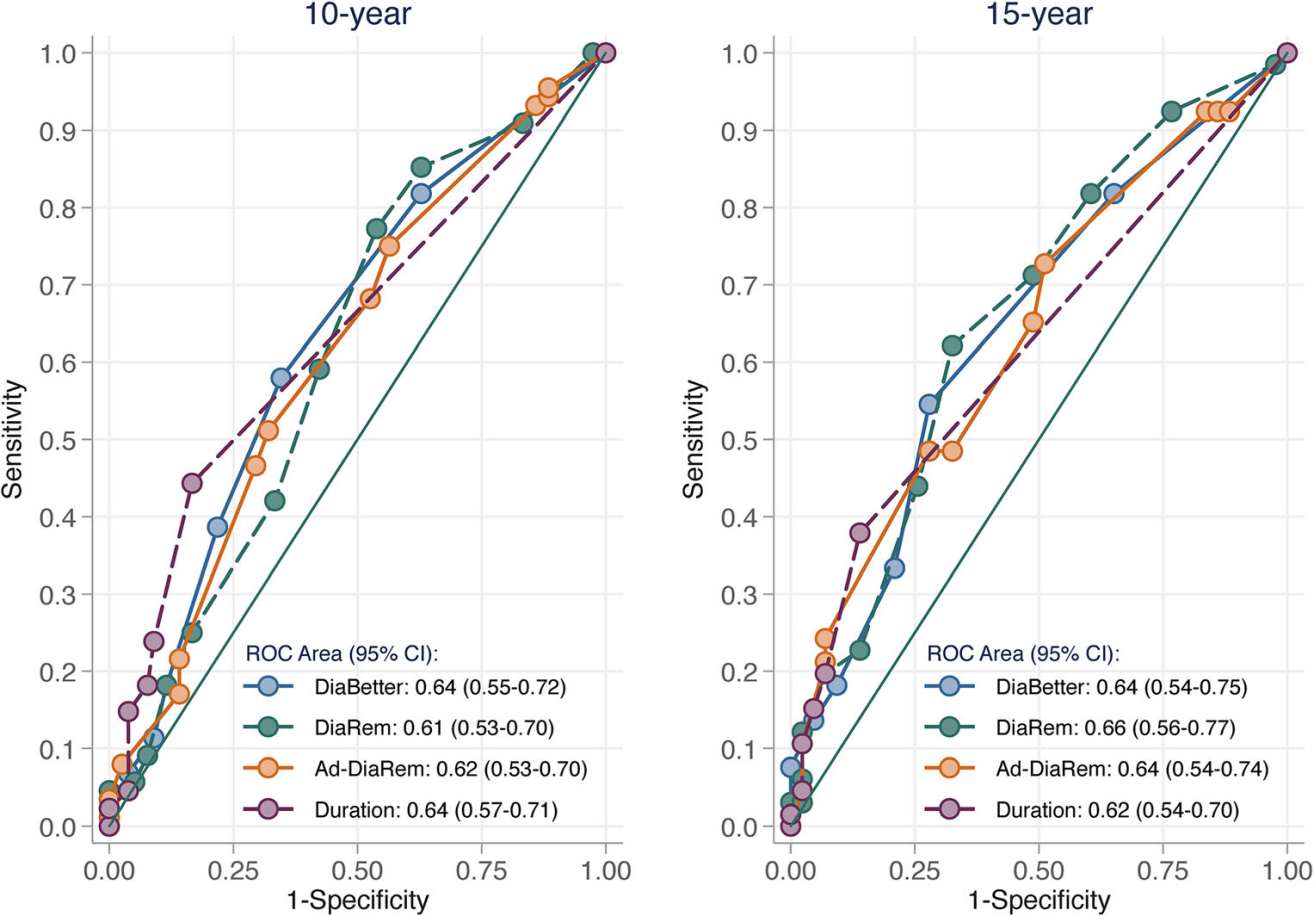
Preoperative prediction of T2D relapse 10 and 15 years after bariatric surgery comparing DiaRem, Ad-DiaRem, and DiaBetter scores, with T2D duration as a single predictor

Predictive capacity in terms of discrimination of all tested models based on preoperative data was poor (Fig. 1). For T2D relapse at 10 years, AUROC curves were 0.61 (95% CI 0.53–0.70) for DiaRem, 0.62 (95% CI 0.53–0.70) for Ad-DiaRem, 0.64 (95% CI 0.55–0.72) for DiaBetter, and 0.64 (95% CI 0.57–0.70) for T2D duration alone. For T2D relapse at 15 years, the corresponding AUROC curves ranged between 0.62 and 0.66. There was no difference in predictive capacity between T2D duration alone and any of the preoperative scores for T2D relapse between 2 and 10 years or between 2 and 15 years (*p* values between 0.49 and 0.95).

### Postoperative Prediction of Type 2 Diabetes Relapse

**Fig. 2:**
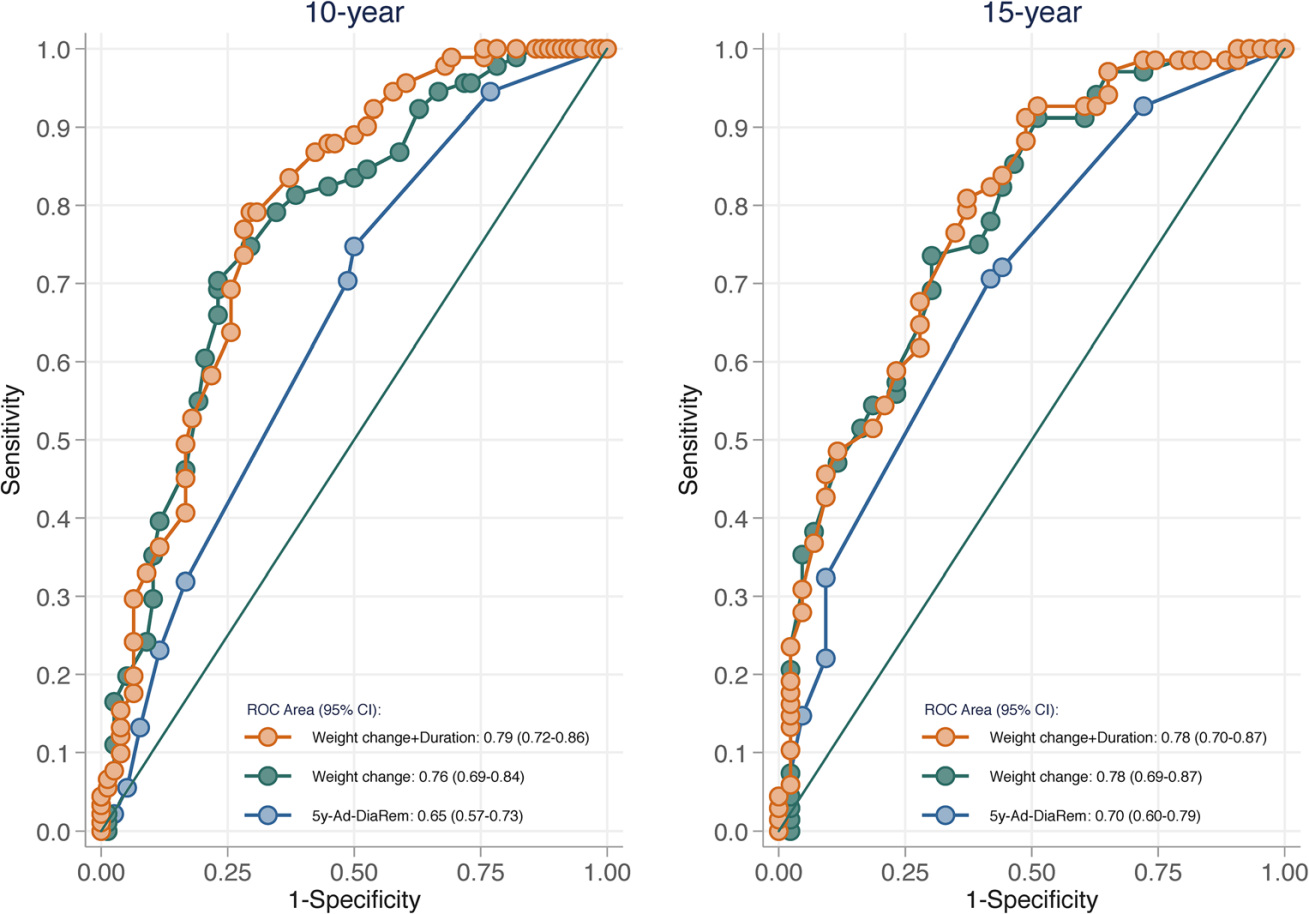
Postoperative prediction of T2D relapse 10 and 15 years after bariatric surgery comparing the 5y-Ad-DiaRem score with 2-year weight change alone or in combination with T2D duration

Inclusion of postoperative measures somewhat increased predictive capacity (Fig. 2). For T2D relapse at 10 and 15 years, AUROC curves were 0.65 (95% CI 0.57–0.73) and 0.70 (0.60–0.79) for 5y-Ad-DiaRem whereas 2-year weight change as a single predictor resulted in AUROC curves of 0.76 (0.69–0.84) for 10-year relapse and 0.78 (0.69–0.87) for 15-year relapse, respectively.

The model with 2-year weight change as a single predictor displayed higher discriminatory capacity than the composite 5y-Ad-DiaRem score for 10-year relapse (*p* = 0.036). For 15-year relapse, 2-year weight change alone had similar discriminatory capacity as the 5y-Ad-DiaRem score (*p* = 0.188). Predictive capacity was not significantly improved by combining 2-year weight change with baseline T2D duration in the 10-year or 15-year relapse models (Fig. 2).

The 5y-Ad-DiaRem model is originally based on 1-year weight change data [12]. In a sensitivity analysis, we therefore analyzed the discriminatory capacity of 1-year weight change as a single predictor. Both at 10 and 15 years, there was a trend that 1-year weight change had higher predictive capacity than the 5y-Ad-DiaRem. Moreover, 2-year weight change (AUROC 0.76) had higher predictive capacity than 1-year weight change (AUROC = 0.71; *p* = 0.032) for 10-year relapse prediction (Supplementary Table 3).

## Discussion

Our post hoc analysis of participants in the SOS study with preoperative T2D who underwent bariatric surgery shows that all tested models have limited discriminatory power to identify relapse after initial remission. Inclusion of postoperative predictors only marginally increased prediction accuracy. However, 2-year weight change alone had higher discriminatory capacity than the 5y-Ad-DiaRem score for 10-year relapse and similar capacity at 15 years.

If patients with high risk of relapse could be identified before, or soon after the surgical intervention, they may be targeted for intensified postoperative management to reduce the risk of T2D relapse, or benefit from un-delayed initiation of T2D treatment, once re-diagnosed. Accurate clinical prediction models for T2D relapse after initial remission could therefore increase the likelihood for durable remission and reduce the long-term risk of serious T2D complications [2, 17]. However, our results confirm previous observations suggesting that the ability to predict T2D relapse after initial remission using baseline data is limited [11, 12]. For all preoperative scores, and prediction using baseline T2D duration alone, AUROC curves were below 0.65, indicating poor discrimination between those who will and will not relapse. It has been suggested that discrimination can be significantly improved by adding postoperative measures [12]. However, in the current report, the 5y-Ad-DiaRem postoperative scoring model only marginally increased discriminatory capacity and 2-year weight change alone displayed significantly higher or similar predictive capacity at the 10-year and 15-year follow-up, respectively. This finding is in line with other studies showing that weight loss is an independent predictor of T2D outcome 2–5 years after surgery [11, 12] and our previous results from the SOS study demonstrating that weight loss is important for glucose control in the long term [2, 18]. It should be noted that the SOS study contains a high proportion of patients with screen-detected T2D, resulting in a low average T2D duration, which may explain why addition of T2D duration to the 2-year weight change model did not increase the discriminatory capacity. However, the difference in T2D duration in non-relapse (00.4 and 0.3 years, respectively) and relapse (1.1 and 0.9 years, respectively) groups at 10 and 15 years confirms the importance of early surgical intervention for long-term glycemic control [2, 19, 20].

Important strengths of the SOS study are the very long follow-up and the prospective study design. Limitations of the SOS study include the use of older surgical techniques; the majority of patients in the surgery group underwent vertical banded gastroplasty or banding and only a smaller proportion underwent gastric bypass, thus preventing subgroup analyses due to lack of power. It should be noted that most prediction models were developed using short-term data from gastric bypass patients although some studies indicate that both the DiaRem and Ad-DiaRem scores are useful tools for short-term prediction of T2D remission also in patients treated with sleeve gastrectomy and gastric banding [5, 21]. Furthermore, the SOS study was initiated before repeated measurements were routinely used for the diagnosis of T2D and diagnosis is therefore based on analyses of blood samples from a single time point. In addition, there was a large proportion of screen-detected T2D in our study. Thus, similar analyses in larger cohorts with a higher variability of T2D duration and larger number of patients treated with modern surgical techniques such as gastric bypass or sleeve gastrectomy will be needed to assess generalizability of our results.

In conclusion, our results from the SOS study confirm that early weight change after bariatric surgery is a key predictor for T2D relapse and suggest that composite prediction models have limited additional value. Intensified postoperative management may be warranted in patients with limited weight reduction.

## Electronic Supplementary Material

ESM 1 (DOCX 51 kb).
