# Supplementary material for: Evaluation of Prediction Models for Type 2 Diabetes Relapse After Post-bariatric Surgery Remission: a Post hoc Analysis of 15-Year Follow-up Data from the Swedish Obese Subjects (SOS) Study
Source: Obes Surg. 2020 Jun 13;30(10):3955–60. doi: 10.1007/s11695-020-04763-2 (PMC7467912; doi:10.1007/s11695-020-04763-2)
Supplement: Supplementary file 1 — (DOCX 51 kb). [file 11695_2020_4763_MOESM1_ESM.docx]

# Electronic supplementary material

**Evaluation of prediction models for type 2 diabetes relapse after post-bariatric surgery remission: a post-hoc analysis of 15-year follow-up data from the Swedish Obese Subjects (SOS) study**

| **Table of contents** | **Page** |
| --- | --- |
| Table 1. The SOS study- inclusion and exclusion criteria | 2 |
| Table 2. Analysed prediction models | 3 |
| Table 3. Postoperative prediction (comparison with 1-year weight change) | 5 |
| References | 6 |

## **Table 1.** The SOS study- inclusion and exclusion criteria

| **Inclusion** | **Exclusion** |
| --- | --- |
| Age 37-60 years | Earlier surgery for gastric or duodenal ulcer |
| BMI ≥34 kg/m^2^ in men and ≥38 kg/m^2^ in women | Earlier bariatric surgery |
|  | Gastric ulcer during the past 6 months |
|  | Ongoing malignancy or active malignancy  during the past 5 years |
|  | Myocardial infarction during the past 6 months |
|  | Bulimic eating pattern |
|  | Drug or alcohol abuse |
|  | Psychiatric or cooperative problems contraindicating bariatric surgery |
|  | Other contraindicating conditions^^[[1]](#footnote-1)^^ |

## **Table 2.** Analysed prediction models

| **Pre-surgery prediction factor** | **DiaRem (1)** | |  | **Ad-DiaRem (2)** | |  | **DiaBetter (3)** | |  | **5y-Ad-DiaRem (4)** | |
| --- | --- | --- | --- | --- | --- | --- | --- | --- | --- | --- | --- |
| Age (y) | <40 | 0 |  | 15-41 | 0 |  |  |  |  |  | - |
|  | 40-49 | 1 |  | 42-52 | 3 |  |  |  |  |  | - |
|  | 50-59 | 2 |  | 52-69 | 5 |  |  |  |  |  | - |
|  | ≥60 | 3 |  |  |  |  |  |  |  |  | **-** |
| HbA1C mmol/mol  (%) | <48  (6.5) | 0 |  | 26-52  (4.5-6.9) | 0 |  | ≤48 (6.5) | 0 |  | <45 (6.3) | 0 |
|  | ≥48–≤52 (6.5-6.9) | 2 |  | 53-57 (7.0-7.4) | 2 |  | 49-55 (6.6-7.2) | 1 |  | 45-<52 (6.3-6.9) | 1 |
|  | ≥53-≤74 (7.0-8.9) | 4 |  | 58 -178 (7.5-18.4) | 4 |  | 56–68 (7.3-8.4) | 2 |  | ≥52 (6.9) | 3 |
|  | ≥75 (9.0) | 6 |  | - | - |  | ≥69 (8.5) | 3 |  |  |  |
| Insulin treatment | No | 0 |  | No | 0 |  | No | 0 |  |  | - |
|  | Yes | 10 |  | Yes | 3 |  | Yes (alone or in combination) | 3 |  |  | - |
| Metformin | Only metformin | 0 |  | Only metformin | 0 |  | Only metformin | 1 |  |  | - |
| Other anti-diabetic drugs | SU and insulin sensitizing agents other than metformin | 3 |  | Other medications^e^ | 1 |  | Only other non-insulin drug(s) | 2 |  |  | - |
| No. of medications^b,c^ |  | - |  | 0 | 0 |  |  | - |  | 0 | 0 |
|  |  | - |  | 1 | 1 |  |  | - |  | 1 | 1 |
|  |  | - |  | 2 | 2 |  |  | - |  | 2 | 3 |
|  |  | - |  | ≥3 | 3 |  |  | - |  | ≥3 | 4 |
| Duration (years) | ≤5 | - |  | 0-6.9 | 0 |  | ≤2 | 0 |  | <1 | 0 |
|  | 6-9 | - |  | 7-13.9 | 3 |  | 2.1-5.0 | 1 |  | 1-<3 | 1 |
|  | ≥10 | - |  | ≥14 | 5 |  | 5.1-10.0 | 2 |  | 3-<5 | 2 |
|  |  | - |  | - |  |  | ≥10.1 | 3 |  | 5-<7 | 3 |
|  |  |  |  |  |  |  |  |  |  | ≥7 | 4 |
| **Post-surgery prediction factor** **^a^** | **DiaRem (1)** | |  | **Ad-DiaRem (2)** | |  | **DiaBetter (3)** | |  | **5y-Ad-DiaRem (4)** | |
| No. of medications |  | - |  |  | - |  |  | - |  | 0 | 0 |
|  |  | - |  |  | - |  |  | - |  | 1 | 1 |
|  |  | - |  |  | - |  |  | - |  | ≥2 | 4 |
| Fasting blood glucose (mmol/l) |  | - |  |  | - |  |  | - |  | <4.8 | 0 |
|  |  | - |  |  | - |  |  | - |  | 4.8-<5.3 | 1 |
|  |  | - |  |  | - |  |  | - |  | 5.3-<5.8 | 2 |
|  |  | - |  |  | - |  |  | - |  | ≥5.8 | 3 |
| Body weight lost from baseline (%)^f^ |  | - |  |  | - |  |  | - |  | <-34 | 0 |
|  |  | - |  |  | - |  |  | - |  | -34-<-25 | 1 |
|  |  | - |  |  | - |  |  | - |  | -25-<-20 | 2 |
|  |  | - |  |  | - |  |  | - |  | ≥-20 | 3 |
| Remission status^g^ |  | - |  |  | - |  |  | - |  | DR | 0 |
|  |  | - |  |  | - |  |  | - |  | PDR | 3 |
|  |  | - |  |  | - |  |  | - |  | NDR | 5 |
| **Range of score** |  | **0-22** |  |  | **0-21** |  |  | **0-9** |  |  | **0-26** |

^a^ Original 5y-Ad-DiaRem score developed using post-surgery data 1 year after bariatric surgery. For analyses in the SOS study only 2-year data were available.

^b^ Ad-DiaRem: Includes sulfonylureas, insulin sensitising agents and GLP-1 analogues, DDP-IV inhibitors, insulin and other glucose-lowering agents

^c^ 5y-Ad-DiaRem: Includes all antidiabetic treatments, regardless of pharmacological class (insulin included).

^d^ Conditional depending on type 2 diabetes duration.

^e^ Includes sulfonylureas (glimepiride, glipizide and glibenclamide), insulin sensitising agents other than metformin (pioglitazone and rosiglitazone).

^f^ The weight loss was calculated using the following formula: [(2-year body weight)-(baseline body weight)]/(baseline body weight)x100

^g^ DR, diabetes remission (HbA1c<39 mmol/mol (5.7%) or blood glucose <5.0 mmol/L (plasma glucose <5.6 mmol/L).; PDR, partial diabetes remission (HbA1c <48 mmol/mol or blood glucose concentration <6.1 mmol/L (plasma glucose <7 mmol/L and no diabetes medication), NDR, non-remission (HbA1c ≥48 mmol/mol (6.5%) or blood glucose of ≥6.1 mmol/L (plasma glucose ≥7 mmol/L), or diabetes medication.

## **Table 3.** Postoperative prediction using the 5y-Ad-DiaRem score, compared with 1- or 2-year weight change alone or in combination with type 2 diabetes duration.

| **Relapse predicted** | **5y-Ad-DiaRem** | **1-year weight change** | | **2-year weight change** | | | **2-year weight change + duration** | | |
| --- | --- | --- | --- | --- | --- | --- | --- | --- | --- |
|  | AUROC | AUROC | p^a^ | AUROC | p^a^ | p^b^ | AUROC | p^a^ | p^c^ |
| 10-year follow-up | 0.65 (0.57-0.73) | 0.71 (0.63-0.79)^d^ | 0.331 | 0.76 (0.69-0.84) | **0.036** | **0.032** | 0.79 (0.72-0.86) | **0.002** | 0.209 |
| 15-year follow-up | 0.70 (0.60-0.79) | 0.75 (0.66-0.84)^e^ | 0.437 | 0.78 (0.69-0.87) | 0.188 | 0.340 | 0.78 (0.70-0.87) | 0.113 | 0.914 |

^a^ Comparison with the 5y-Ad-DiaRem composite score.

^b^ Comparison with 1-year weight change.

^c^ Comparison with 2-year weight change.

^d^ The relative weight change (mean (SD)) at 1 year in the non-relapse and relapse group was -29.4 (9.8) % and -22.8 (7.8) %, respectively (3 missing).

^e^ The relative weight change (mean (SD)) at 1 year in the non-relapse and relapse group was -30.6 (9.1) % and -22.7 (8.5) %, respectively (0 missing).

### **References**

1. Still CD, Wood GC, Benotti P, Petrick AT, Gabrielsen J, Strodel WE, et al. Preoperative prediction of type 2 diabetes remission after Roux-en-Y gastric bypass surgery: a retrospective cohort study. The lancet Diabetes & endocrinology. 2014 Jan;2(1):38-45.

2. Aron-Wisnewsky J, Sokolovska N, Liu Y, Comaneshter DS, Vinker S, Pecht T, et al. The advanced-DiaRem score improves prediction of diabetes remission 1 year post-Roux-en-Y gastric bypass. Diabetologia. 2017 Oct;60(10):1892-902.

3. Pucci A, Tymoszuk U, Cheung WH, Makaronidis JM, Scholes S, Tharakan G, et al. Type 2 diabetes remission 2 years post Roux-en-Y gastric bypass and sleeve gastrectomy: the role of the weight loss and comparison of DiaRem and DiaBetter scores. Diabetic medicine : a journal of the British Diabetic Association. 2018 Mar;35(3):360-7.

4. Debédat J, Sokolovska N, Coupaye M, Panunzi S, Chakaroun R, Genser L, et al. Long-term Relapse of Type 2 Diabetes After Roux-en-Y Gastric Bypass: Prediction and Clinical Relevance. Diabetes care. 2018 Oct;41(10):2086-95.

1. Such as chronic glucocorticoid or anti-inflammatory treatment [↑](#footnote-ref-1)
